# Malaria control under the Taliban regime: insecticide-treated net purchasing, coverage, and usage among men and women in eastern Afghanistan

**DOI:** 10.1186/1475-2875-9-7

**Published:** 2010-01-06

**Authors:** Natasha Howard, Ahmad Shafi, Caroline Jones, Mark Rowland

**Affiliations:** 1 London School of Hygiene and Tropical Medicine, London, UK; 2 HealthNet-TPO Malaria & Leishmaniasis Control Programme, Kabul, Afghanistan

## Abstract

**Background:**

Scaling up insecticide-treated mosquito net (ITN) coverage is a key malaria control strategy even in conflict-affected countries [1,2]. Socio-economic factors influence access to ITNs whether subsidized or provided free to users. This study examines reported ITN purchasing, coverage, and usage in eastern Afghanistan and explores women's access to health information during the Taliban regime (1996-2001). This strengthens the knowledge base on household-level health choices in complex-emergency settings.

**Methods:**

Fifteen focus group discussions (FGDs) and thirty in-depth interviews were conducted with men and women from ITN-owning and non-owning households. FGDs included rank ordering, pile sorting and focused discussion of malaria knowledge and ITN purchasing. Interviews explored general health issues, prevention and treatment practices, and women's malaria knowledge and concerns. Seven key informant interviews with health-related workers and a concurrent survey of 200 ITN-owning and 214 non-owning households were used to clarify or quantify findings.

**Results:**

Malaria knowledge was similar among men and women and ITN owners and non-owners. Women reported obtaining health information through a variety of sources including clinic staff, their husbands who had easier access to information, and particularly female peers. Most participants considered ITNs very desirable, though not usually household necessities. ITN owners reported more household assets than non-owners. Male ITN owners and non-owners ranked rugs and ITNs as most desired, while women ranked personal assets such as jewellery highest. While men were primarily responsible for household decision-making and purchasing, older women exerted considerable influence. Widow-led and landless households reported most difficulties purchasing ITNs. Most participants wanted to buy ITNs only if they could cover all household members. When not possible, preferential usage was given to women and children.

**Conclusions:**

Despite restricted access to health facilities and formal education, Afghan women were surprisingly knowledgeable about the causes of malaria and the value of ITNs in prevention. Inequities in ITN usage were noted between rather than within households, with some unable to afford even one ITN and others not wanting ITNs unless all household members could be protected. Malaria knowledge thus appears a lesser barrier to ITN purchasing and coverage in eastern Afghanistan than are pricing and distribution strategies.

## Background

Scaling up coverage of insecticide-treated nets has become a global malaria-control strategy [1,2]. Many countries, striving to reach malaria-related Millennium Development Goal (MDG) and Roll Back Malaria (RBM) targets, rely on ITN implementation [3,4]. However, socio-economic factors can heavily influence success, even with free ITN or long-lasting insecticidal net (LLIN) distribution [5-8]. ITN purchasing can depend on cost and availability, perceived value and safety, ideas of disease causation, risk conceptions, and peer acceptance [8-10]. Regular usage can depend on amount of insect nuisance biting, perceived malaria danger, sleeping patterns, comfort, and convenience considerations [7,11,12]. This study examines aspects of reported ITN purchasing, coverage and usage in eastern Afghanistan during Taliban control. By disaggregating women's malaria knowledge and reported behaviour from that of men, it builds on limited knowledge of household-level health choices in socially conservative complex-emergency settings [5-7,13-16].

Before Afghanistan's extended conflict, malaria was almost eliminated as a public health issue through vertical governmental indoor residual spraying (IRS) and chloroquine treatment [17-19]. As control infrastructure deteriorated malaria rates increased, peaking during the mid-1990s [17,19]. From 1992, eastern Afghanistan became stable enough to establish a network of NGO-supported clinics, standardize training and monitoring of microscopists and clinical staff, and distribute ITNs and insecticide retreatment. Malaria control was coordinated by technical agency HealthNet-TPO (HNI-TPO) [19]. During the mujahedeen and Taliban eras (1992-96 and 1996-2001), HNI-TPO developed a package of interventions shown through operational research to be effective, popular and not requiring significant government input [17,18,20-24]. Government infrastructure and support remained minimal [25].

HNI-TPO has coordinated wide promotion and subsidized sale of ITNs in eastern Afghanistan and refugee communities in Pakistan since 1992 [19]. Endemicity is seasonal and health infrastructure still weak, making household-level interventions more practicable and cheaper for implementing agencies [17]. Using several distribution strategies, HNI-TPO disseminated sufficient family-size ITNs to cover approximately one million Afghans by the time the Taliban first gained power. Strategies included mobile teams of health educators and logisticians for remote areas, community-based ITN implementers, and clinic-based health education, sales and insecticide treatment provided by trained clinic staff. By 2000, HNI-TPO was attempting cost recovery to create a revolving fund that could extend the limited resources then available for malaria control [3,19,26,27].

Investigators were not convinced that coverage achieved by these strategies could provide sufficient community-wide protection, and wanted to find ways to increase ITN purchasing. As part of this, social research was undertaken in 2000 among men and women from ITN-owning and non-owning households in Nangarhar Province, Eastern Afghanistan. A concurrent socio-economic analysis showed that the wealthiest 25% of households surveyed were 4.5 times more likely to own ITNs than the poorest 25% [9]. Objectives of qualitative data collection were to deepen understanding of factors affecting ITN purchasing constraints, coverage, and usage patterns in the area.

## Methods

### Target population and study design

**Figure 1:**
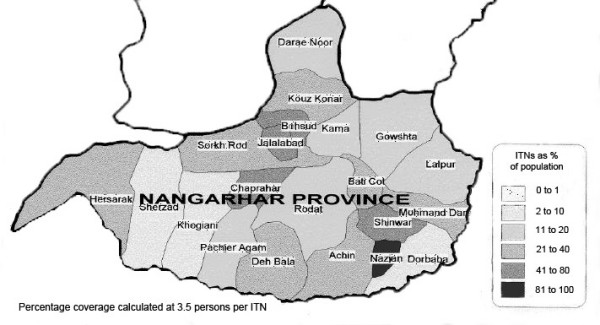
**Afghanistan's Nangarhar Province and districts, showing percentage ITN coverage**. Source: HN-TPO, Jalalabad office 2000.

The target population was men and women in ITN-owning and non-owning households from an estimated population of 500,000 in Nangarhar Province exposed to HNI-TPO's ITN programme (Figure 1). Study design incorporated focus group discussions (FGDs), in-depth interviews, and a quantitative household survey. Study components were conducted from February to September 2000. Inclusion of low and high transmission seasons was intended to help reduce overestimation of malaria risk and ITN usage. 'ITN ownership' was defined as having one or more ITNs. 'ITN usage' was defined as 'sleeping under an ITN,' rather than the narrower 'sleeping under an ITN the previous night' sometimes used in the literature, as ITN usage reflected mosquito and malaria seasonality and research indicated owners actually used their ITNs [18,24]. 'Household' was defined as a family group sharing a compound and cooking facilities. Ethical approval was granted by the London School of Hygiene & Tropical Medicine (LSHTM) Research Ethics Committee.

FGDs were conducted separately with men and women from ITN-owning and non-owning households. Sampling was purposive with the main aim to determine the feasibility of conducting community-level social research in this setting. Following initial analysis of FGDs, the in-depth interviews and household survey were conducted. Sampling was multi-stage. First, eight districts were purposefully selected to include those regularly targeted by ITN sales campaigns and those further from Jalalabad, the provincial capital, that had been targeted less frequently. Second, approximately twenty-four villages were randomly selected from those previously exposed to HNI-TPO's ITN campaigns. Third, households were randomized numerically. Thirty semi-structured interviews, again arranged separately for men and women from ITN-owning and non-owning households, were conducted concurrently with 400 household survey interviews. Survey sample size was calculated to detect a difference of 10% versus 20% between strata of equal size with 80% power and 95% confidence interval. The questionnaire was developed from similar instruments and FGD results, and piloted in three villages not included in the study. In-depth interview sampling was a mix of theoretical and convenience as described by Green and Thorogood, with approximately four interviews conducted per district (i.e. one man and one woman from different ITN-owning and non-owning households) [28]. Seven key informant interviews were completed with clinic staff, informal health providers, and ITN implementers on a convenience basis to clarify findings. Informed consent was obtained from all participants and data anonymized.

### Data collection and analysis

FGDs were conducted during February in Achin, Muhmand-Dara and Shinwar, three districts selected for accessibility, security, and ITN ownership of less than 40%. Each was moderated in Pashtu or Dari by a trained local researcher of the same gender as participants and supervised by an experienced LSHTM-based qualitative researcher. FGDs took place in locations selected by participants and were tape-recorded, with participants' verbal consent, for later transcription and translation. FGDs included formal pile-sorting and rank-ordering exercises and targeted discussion of malaria knowledge and perceptions, ITNs and retreatment, and gender and cultural attitudes.

In-depth interviews were conducted in March-April in Achin, Bati Kote, Bihsood (Jalalabad), Lalpur, Muhmand-Dara, Nazyan, Shinwar, and Surkhrod districts, by a male and female researcher using a sub-headed topic guide. Interviews investigated FGD topics plus perceptions of malaria's relative importance, prevention and treatment practices, and women's malaria-related health information and access. All, except three with doctors in English, were interpreted directly between English and Pashtu or Dari. Authors were constrained by the lack of an experienced female interpreter, but recruited a male researcher who appeared youthful enough to be considered culturally acceptable. Researchers chose not to tape interviews because several male family members refused consent for women to be recorded and researchers wanted to interview as many women as possible and remain methodologically consistent [29,30]. To minimize data loss, both researchers took notes under simple topic codes in English or Dari/Pashtu. Notes and observations were discussed by researchers after each interview and written up each evening [30].

Structured household interviews were conducted in Pashtu or Dari by five trained male data collectors in twenty-one villages in the eight districts. The questionnaire included demographics, socio-economics, health knowledge, malaria, mosquitoes, and ITNs. Socio-economic findings and more detailed methodology and analysis are published elsewhere [9].

Data were transcribed and translated in Peshawar and analysed in London. The full range of responses and views expressed by FGD and interview participants was assessed and shared responses grouped using qualitative content analysis with manual thematic coding [31]. Survey data was analysed in Stata^® ^and outcomes assessed for associations with ITN ownership using logistic regression.

## Results

Fifteen FGDs were conducted in six villages; five with male ITN owners, one with female ITN-owners, five with male non-owners, and four with female non-owners. Eighty-two men and approximately 40 women participated. Rank-ordering and pile-sorting exercises were successfully completed in men's but not women's FGDs owing to moderator inexperience; thus pile-sorting and ranking analysis is restricted to data from men's FGDs.

Thirty in-depth interviews were conducted in eight districts; eight with men and six with women from ITN-owning households, and seven with men and eight with women from non-owning households. Most women chose to be interviewed with female relatives or friends present, due to the absence of a female interpreter. Seven key informant interviews, completed with health-related workers (i.e. three doctors, one laboratory technician, one traditional healer, and two ITN implementers), were analysed separately.

Two-hundred ITN-owning and 214 non-owning households completed survey interviews. The response rate was 95%, with non-participation reported as due to ongoing poppy harvesting. All but five household heads were male, and only 48 (11.6%) respondents were female. Most self-identified as Pashtun, while 36% in Bihsood district were of Tajik ethnicity. Half of households (58%) relied on agriculture. Main crops were wheat, opium poppy, corn, and cotton. Most (83%) came to the area in 1993, when security improved in eastern Afghanistan.

### Pile sorting and ranking exercises

Information generated by pile sorting indicated differences in household asset ownership between participating ITN owners and non-owners. Non-owners generally reported fewer possessions than ITN owners. This was particularly noticeable for rugs (59% versus 27%), radios (54% versus 29%), jewellery (54% versus 29%) and pressure cookers (54% versus 29%). Some non-owners reported very few household possessions. For example, 16% reported no kettle and 12% no lamps, whereas all ITN owners had these items. Approximately 15% of non-owners reported similar quantities of household assets as ITN owners (i.e. radio plus at least two - rug, pressure cooker, or jewellery). This reinforced quantitative survey findings, that households with at least one ITN were likely to have more assets than those without ITNs [9].

FGD participants were asked to rank household assets in the order in which they would most likely procure them if they had extra cash or goods for bartering. Rugs and ITNs ranked highest among both ITN owners and non-owners. Items deemed essential, such as clothes and a lamp, ranked in the top three for non-owners. Bicycles and pressure cookers, reported as luxury items, were ranked in the top three by ITN owners. In discussing ranking, non-owners explained that they ranked clothing highly because it was essential for wives to keep covered when going outside due to Taliban restrictions. They also revealed that rugs, topping the list for both groups, were seen as status items. Even men who already owned rugs said they would, as a first choice, purchase more. ITN ownership was also described as reflecting status, though data needs caution as FGD participants were aware that researchers were associated with HNI-TPO's malaria control programme. However, both ITN owners and non-owners ranked indoor residual spraying (IRS) highly, suggesting that mosquitoes and other insects were problematic.

Women were eager to participate in FGD discussions, but appeared so determined to talk that they generally did not complete formal exercises. Discussions clarified that women ranked clothing and jewellery, assets Afghan women own personally, as the two most important household assets.

### Knowledge and reported behaviour

Interview, survey, and remaining FGD results are reported under key themes: malaria knowledge and perceptions; malaria prevention and treatment; ITN knowledge and perceptions; reported ITN purchasing; reported ITN coverage and usage; and health-related workers' perceptions.

#### Malaria knowledge and perceptions

There appeared to be little difference in knowledge of malaria transmission between genders or between ITN owners and non-owners. Table 1 shows approximately 75% of survey respondents said mosquitoes caused malaria, though 19% said it was caused by water. Unsurprisingly, participants could not distinguish between vector and nuisance mosquitoes.

**Table 1:** Percentage reported malaria knowledge and practices, comparing ITN-owning to non-owning households

	% ITN Non-owners (n = 214)	% ITN Owners (n = 200)
*What causes malaria?*		
Mosquitoes	77	73
Water	18	20
Other/Don't know	6	8

*Where do mosquitoes breed?*		
Water	58	69
Grass	23	20
Other/Don't know	19	12

*Malaria season*		
Summer	46	40
Spring/Summer	33	39
Autumn	11	11
Other/All	10	10

*Who is at most risk from malaria?*		
Children	50	48
Women and children	25	19
Everyone	21	17
Pregnant women and under-fives*	1	10
Women	3	4
Aged	1	2

What is the best malaria protection?*		
ITNs	74	86
IRS	12	7
Other (e.g. electric fans)	7	5
Traditional	3	1
Don't know	4	1

What current malaria protection do you use?*^1^		
ITNs	4	95
Other (e.g. smoke, chadors)	92	1
Insecticide spray	4	3
Traditional	0	1

*Who in your household was seriously ill this year?*		
All	57	54
Children	35	32
None	4	8
Aged	3	3
Women	1	3

What is the best treatment for malaria?*		
Chloroquine	68	79
Don't know	21	11
Traditional/Other	7	7
Paracetamol	4	3

*Who makes treatment-seeking decisions?*		
Household head	92	90
Other	8	10

*Where do you go for malaria treatment?*		
Get treatment at NGO clinic	42	48
Private doctor (unregulated)	35	24
Other/Combination	22	28
Private drug seller (unregulated)	2	1

*Average reported costs for malaria treatment*^2^	*(US$08)*	*(US$08)*
Adult visit	0.58	0.44
Adult drugs	8.19	5.98
Child visit	0.56	0.42
Child drugs	5.25	3.89

*Average costs per ITN*^2^	*(US$08)*	*(US$08)*
ITN (insecticide added at point-of-purchase)	6.50	6.50
ITN retreatment (annual)	0.07	0.07

**NB**: Sample size is 414. Logistic regression *p-value < 0.05 or ^1^p < 0.001. ^2^Costs were not disaggregated by provider. Pakistani rupee 2000 prices have been converted to US dollar 2008 constant equivalents (US$08).

"When mosquitoes bite healthy people they catch malaria." (Female non-owner, Ghazgay, Muhmand-Dara)

Approximately one-third of interviewees suggested mosquito bites were only one way of catching malaria with the main contributor reported as drinking dirty water. Most survey respondents (64%) knew that mosquitoes breed in water, but most participants did not know how mosquitoes transmit malaria, often describing faecal-oral routes (e.g. mosquitoes breed in dirty water and garbage, and this dirt infects people when they are bitten).

"Malaria is caused by mosquitoes who get parasites from dirty water" (Male ITN owner, Muhmand-Dara)

Another belief reported in each district was that malaria, if it continues or increases in severity, becomes *moriqa *(typhoid).

"Malaria comes from mosquitoes and dirty water. Mosquitoes breed in dirty ponds, cow dung and refuse. malaria becomes typhoid if it is not cured" (Male non-owner, Bati Kote)

"The clinic doctors told us typhoid is from malaria." (Female ITN owner, Ghani Khel, Shinwar)

Participants reported that malaria was a serious illness. In interviews, researchers attempted to estimate its perceived importance by asking participants the three greatest health concerns in their community. The top three concerns reported were diarrhoea, 'maternal problems,' and tuberculosis.

While not directly related to this research, it is interesting that as early as 2000, maternal ill health was reported as a major concern by both genders in all districts. It was described as a particular concern due to female travel restrictions, and lack of female health staff or culturally acceptable facilities. Several women mentioned the lack of confidential contraception as their main health concern.

"It's not appropriate for our women to give birth publicly in the clinic. Many women have serious problems during childbirth. We can't afford when the lady doctor comes to the house and anyway she usually doesn't because she can't come alone. There is no one around who knows how to birth the baby properly and so many die."(Male non-owner, Bati Kote)

Health messages, aimed at men for cultural reasons, did appear to reach women. Women interviewees reported getting most of their health and ITN information from clinics, their husbands, or most commonly from each other.

"When one of us learns something, then she tells it to the others." (Female non-owner, Shinwar)

"Our husbands don't let us listen to radio because it uses up the batteries. We are encouraged to listen to religious programmes. I learned about ITNs from my husband and the clinic doctors." (Female ITN owner, Nazyan)

#### Malaria prevention and treatment

Eighty percent of survey respondents said ITNs were the best means of malaria prevention. Interviewees reported that other common forms of protection against mosquitoes were burning grass, rubbing lamp or motor oil on the skin, and sleeping wrapped in wet *chadors *(traditional outer garments).

"Preventive measures are good against malaria, but there are no effective ones; sprays wash off, ITNs only protect part of the time. Burning straw is very effective against mosquitoes, but there is some problem with coughing and TB. Electricity is better because we can use fans." (Female non-owner, Shinwar)

Many participants said previous government IRS campaigns had been very effective against mosquitoes, though only 9% of survey respondents considered IRS to be the best means of malaria prevention. These IRS campaigns were generally described as intrusive, but most said they favoured a return to spraying. IRS provision had been free and sprayers reportedly paid for information on households with malaria cases. Now households needed to spend their own money on ITNs.

"We are talking about 20 years ago when the government authorities were spraying houses by force. We didn't know the benefits of spraying and now we know how effective it was!" (Male ITN owner, Pakhail, Achin)

"Spraying should be done by the government, because if we spray individually or have ITNs, mosquitoes will keep coming from our neighbours' houses." (Female non-owner, Bihsood)

Most participants, including 73% of survey respondents, named chloroquine as the best treatment for malaria. Some interviewees, though only 7% of survey respondents, favoured traditional treatments The main reason reported was cost, though a minor percentage were concerned about safety (e.g. for pregnant women). Traditional treatments included cooling drinks, such as *dogh *or *lassie *(from yoghurt), or various plants, the most common of which was a tea from *shamaki *roots. *Shamaki *is a Pashtu term for a plant used locally in traditional medicine, said by some respondents to contain quinine.

"We usually resort to traditional treatment rather than clinical treatment unless the traditional treatment doesn't work. It is due to poverty and people can't afford to cover doctor's and transportation costs." (Male ITN owner, Gharzay, Muhmand-Dara)

Some interviewees said they would only buy half the recommended tablets to reduce treatment costs, while others reported they could be treated on credit.

"...sometimes we borrow from doctors for treating our patient. For instance, doctors in the clinic treat our patient and we will pay them later in the harvest time or as soon as we get cash. The other way is to pay them with wheat or corn." (Male non-owner, Gharzay, Muhmand-Dara)

While participants agreed it was less costly to prevent than to treat malaria, emergency funds for treatment could be borrowed from relatives or neighbours, while funds for protective goods, such as ITNs, could not be readily mobilised.

"I saved money for four years to buy ITNs. I can borrow money from my neighbours and relatives to pay for treatment, but they're not willing to lend for something like a bednet." (Widow, ITN owner, Bihsood)

#### ITN knowledge and perceptions

All participants could accurately describe ITNs and how they should be used. Authors found no differences between genders or ITN-owners and non-owners in recall of health messages about the benefits of sleeping under ITNs.

"Using ITNs has two benefits. One is that it protects you from malaria and the second is that you sleep well." (Male non-owner, Achin)

"We need ITNs for protection against malaria, not for having fun!" (Male non-owner, Achin)

While ITNs were frequently mentioned as playing an important role in the prevention of both nuisance biting and malaria, some participants said they did not want to make the initial investment.

"Malaria is not something that much can be done about, just to endure. ITNs are out of reach and not useful enough to buy." (Male non-owner, Shinwar)

Participants in a women's FGD, asked their views on possible inclusion of ITNs as part of a dowry, responded with laughter. As this differed from men's pile-sorting and ranking results, it indicates that either women valued ITNs less than did men or that ITNs were affordable for many households and thus not of sufficient monetary value to feature in a dowry.

"That is totally absurd! How should we let this stupid boy get married with our daughters by providing us with nets rather than paying?" (Female non-owner, Meydanak, Achin)

"We work hard to bring up our daughter and then to give her for ITNs? It is an absolutely silly thing to do! We are not stupid." (Female non-owner, Meydanak, Achin)

#### Reported ITN purchasing

Table 2 shows 84% of survey respondents said they were planning to buy ITNs. While 57% wanted them to reduce mosquito nuisance, 38% wanted them for malaria protection. Of those not planning to buy ITNs, the primary reasons given were cost (39%) and already having enough (30%). Responsibility for purchasing decisions rested with the household head, almost invariably an adult male - husband, father, or grandfather.

**Table 2:** Percentage reported purchasing intentions, comparing ITN-owning to non-owning households

	% Non-owners (n = 214)	% ITN owners (n = 200)
Planning to buy ITNs*	*89*	*78*
Not planning to buy ITNs	9	19
Not sure about buying ITNs	2	3

We'll buy ITNs when they're available	*81*	*83*
We'll buy ITNs this month	12	12
We'll buy this year/Unknown	7	5

Want ITNs to prevent mosquito bites*	*51*	*62*
Want ITNs to prevent malaria	42	35
Other/Unsure	7	3

Don't want ITNs due to cost*	*46*	*35*
Don't want ITNs due to having enough already	0	47
Other/Unknown*	54	19

**NB**: Sample size is 414. *Logistic regression p-value < 0.05 to < 0.01.

"Head of the family - father or grandfather - is responsible for making the decision to buy something like nets and protection of the family." (Male ITN owner, Hazar Naw, Muhmand-Dara)

Heads of ITN-owning households were significantly better educated. Comparing ITN-owning to non-owning households, household heads with above secondary-school education were 1.85 times more likely to own ITNs than were those with no education (95% confidence interval 1.2-2.8).

Women participants said they had little decision-making power or opportunity to make purchases, but some said their husbands could be persuaded to buy items that they requested. Young women, even if married, were not able to go outside without accompaniment by their husband or parent-in-law.

"We don't go ourselves. Our husbands don't allow us to go for shopping. They usually provide us with what we want them to buy." (Female non-owner, Sunduq, Achin)

"No woman can go anywhere without asking the permission of her husband." (Female ITN owner, Meydanak, Achin)

A common perception of HNI-TPO among interviewees was as an ITN sales company rather than a humanitarian organization. This was despite several local clinics being sign-posted as run by HNI-TPO. However, it was not clear whether this perception was likely to help or hinder HNI-TPO's activities.

Four main purchasing constraints were reported. The first was cost. ITNs were sold for the average equivalent of US$6.50 in 2008 constant prices, with insecticide retreatment costing the current equivalent of US$0.07. Poorest people said they had more urgent problems for daily survival than mosquitoes and fever.

Cost was the most frequently mentioned ITN purchasing constraint among non-owners. Some participants appeared unable to afford an ITN at prevailing prices. Poorest households appeared to be those headed by widows, women whose husbands were disabled or working in Pakistan, and those who did not own enough land to support their household. Women whose husbands were in Pakistan could purchase some supplies from local shops on credit. Credit limits were unclear, though several women said that making ITNs available on credit would increase their ability to buy them.

"ITNs are the best way to protect against malaria, but we can't afford to buy them because we barely have enough to get food every day, and if we have enough for food, we have to buy clothes to cover our bodies. We can't go around naked!" (Female non-owner, Bati Kote)

"We know everything about ITNs but don't have the money to buy." (Male non-owner, Muhmand-Dara)

A second purchasing constraint reported was that participants did not have sufficient money for enough ITNs to cover everyone in their households. Some non-owners expressed reluctance to buy ITNs unless they could provide for the whole household.

"Fifteen people in my family and we have only one net! I don't have any money in hand to buy more nets." (Male ITN owner, Gerday-Ghous, Muhmand-Dara)

"The other problem is that there are 20-30 people in each household and to cover them all with ITNs we need at least 8-10 ITNs that we can't afford to provide." (Male non-owner, Ghazgay, Muhmand-Dara)

A third purchasing constraint, mentioned by both non-owners and owners purchasing additional ITNs, was that seasonal income did not match ITN availability. Several participants complained that ITNs were made available at the beginning of malaria season when they didn't have enough cash to purchase them, and when they did ITNs were no longer available.

"ITNs are available in this village only for a couple of weeks and that's usually the time which doesn't match harvest time (March/April) or when we don't have money" (Male ITN owner, lower Meydanak, Achin)

This lack of consistent availability led some non-owners to speculate that ITN sellers were favouring certain families and health staff were selling ITNs in Pakistan or charging more than they should. However, recent purchasers reported paying the price recommended by HNI-TPO.

"We had money last year, but ITNs were not available. Only relatives and friends of the sellers were able to buy them." (Male, non-owner, Bati Kote)

"Clinic staff sell the ITNs and drugs in the bazaar to make money. If you don't know someone in the clinic, you won't get help." (Female, non-owner, Bihsood)

The final purchasing constraint reported was that perceptions of poor-quality ITN retreatment were discouraging ITN purchasing. Several non-owners reported as a strong purchasing disincentive ITN-owning neighbours telling them ITNs were not as useful as previously. HNI-TPO had recently switched from *permethrin *to *deltamethrin*, and complaints about retreatment with watered down or expired insecticide may have affected ITN sales and retreatment uptake.

"Retreatment is good, but not like it was. Salesmen add more water now, but they say they know what they are doing." (Female ITN owner, Bihsood)

"There has been a gradual decrease in effectiveness since 1994. Maybe the insecticide is not good quality or they're mixing it with too much water. There have been many complaints and many surveys, but nothing ever changes" (Male ITN owner, Bihsood)

"Poor quality retreatment stops people buying ITNs." (Male ITN owner, Pakhail, Shinwar)

#### Reported ITN coverage and usage

Table 3 shows most owners (69%) paid for ITNs from savings. ITN-owning households had an average of three ITNs and four occupants per ITN. Where ITNs were limited, 70% of survey respondents said children and women were given preference. Participants said available ITNs were used by children and women, because they were the weakest and most vulnerable household members and keeping children covered by blankets to protect them from mosquitoes was very difficult. These practices may result from effective health messages, which emphasize the need to cover the most vulnerable (i.e. young children and pregnant women), but also reflect prevailing beliefs that women and children are weak, uninformed and unable to protect themselves.

**Table 3:** Percentage reported ITN usage among ITN-owning households

	% ITN owners (n = 200)
*No. of ITNs per household*	Mean = 2.9 (SD = 2.4)

*Who sleeps under ITNs in your household?*	
Children	36
Women/Children	31
Everyone (sufficient ITNs for all)	29
Women	3
Aged/Other	1

*Are your mosquito nets insecticide treated?*	
Yes	61
No/Unknown	39

*How often are your ITNs retreated?*	
Yearly	78
Bi-annually	12
Don't know/Never	10
After cleaning	1

*Where did you get funds to pay for your ITNs?*	
Savings	69
Loan	17
Gift	8
Crop sales	4
Other	4

**NB**: Sample size is 200.

"They (women and children) are weak in nature and also we men keep covered the exposed parts of our body, though children don't care about this." (Male ITN owner, Meydanak, Achin)

"If malaria mosquitoes bite children they will immediately get ill and can't resist against fever either, and the same applies to women. It is OK with men; they can go to the clinic on foot." (Male ITN owner, Gerday Ghaus)

The average number of children under five per ITN was 1.6 (± SD 1.4), though the number of children under five per household was not associated with ITN ownership (logistic regression p = 0.86). Only one man reported using the household ITN for himself, since as the family bread-winner he wanted to stay healthy. However, a few respondents (1%) said they gave preferential ITN use to the weak old men.

Interviewees reported that most ITN users did not sleep under them throughout the year. The primary reasons given for ITN use were to prevent both nuisance biting and malaria. Malaria was reported to be a more serious but less frequent problem, while nuisance biting was an everyday frustration. Many owners said that they used ITNs only in summer when mosquito densities and nuisance biting were highest and perceptions of malaria risk increased. However, some participants were aware that malaria could be transmitted in other seasons.

"We use ITNs only during the nights and particularly in the summer - only in summer." (Female ITN owner, Ghazai)

"There is (malaria) in winter but not as high as in the summer." (Male ITN owner, Ghazai)

While there was still some risk of both nuisance biting and malaria in winter, several participants said that people slept under blankets in winter so the likelihood of mosquito bites was reduced.

#### Health-related workers' perceptions

Interviews with health workers and ITN implementers supported general findings and sometimes provided additional insight.

Those doctors interviewed considered malaria an important contributor to morbidity and lost productivity, though not the primary disease priority in communities.

"The most dangerous (disease) is TB because it's transmissible easily and also if a person is diseased by this microbe and doesn't take care of himself he will die. But in malaria death is not essential, and the treatment of TB requires more time, at least 6 months, and the drugs are very expensive" (Doctor, Bati Kote)

Almost half of participants reported first going to public/NGO clinics for treatment, because they were cheapest. However, if treatment results were unsatisfactory many also went to private doctors. Several health workers described this type of treatment seeking negatively.

"If the clinic technician says it's not malaria she will think he's no good and go to a private lab where they will tell her it's malaria. There's a lot of overprescribing of chloroquine to make people happy." (Laboratory technician, Bihsood)

While most participants favoured the idea of credit schemes, and said that purchasing ITNs through small weekly or monthly sums would be beneficial, ITN implementers were not so eager:

"I'm only working seven months a year. It would take at least nine months to collect money from credit. We (ITN implementers) know who needs and deserves credit and could take responsibility for monitoring, but they would need to pay us all year round" (ITN implementer, Bihsood)

## Discussion

Many aspects of ITN purchasing are similar for countries during war or peace, under despotic or benign authority. The Taliban, espousing a strict anti-modern antifeminist ideology adapted from Sunni Muslim sharia and Pashtun tribal codes, governed Afghanistan from 1996 to late 2001 [19,32,33]. While the presence of the Taliban placed profound restrictions on mobility, access and acquisition of information, their influence was far from all-pervasive and householders - women in particular - adopted a number of coping strategies to access information and influence health-related family decision-making.

### ITN purchasing

Two key issues influencing purchasing were availability and pricing, constraints also found in several African studies [34-37]. Afghan men and women were aware of the link between mosquitoes and disease and of the protective effects of ITNs. Knowledge did not appear to be the main factor influencing ITN purchase and use, as there was little difference in knowledge between ITN owners and non-owners [38,39]. This is attributable to successful health information from sources such as health facility staff, HNI-TPO implementers, and radio programmes (e.g. BBC Pashtu Service news and dramas). HNI-TPO's ITN coverage reached 60% in some trial areas where no limits were placed on the numbers of ITNs individuals purchased [15]. Overall coverage was around 30% (Figure 1). Among participants who did not have ITNs, most said they wanted but could not afford them. Despite successful transmission of health messages, ITNs were not yet seen as household necessities. Rather, they were one of the first 'extras' families with greater financial stability felt able to afford. Household decision-makers appeared to understand the reasons for having ITNs, but make purchasing decisions based on overall risk perceptions [40,41]. More research is needed to explore this.

Lack of ITN availability when participants said they were ready to purchase suggests delivery strategies could be restructured [42-44]. ITNs were usually available during summer when mosquito nuisance was severe, but spare cash from harvests was already spent. Making ITNs available when people were likely to have cash (e.g. harvest or crop purchase times) could increase sales [44]. Making ITNs more visible throughout the year (e.g. through mobile salesmen or local shops) could improve awareness of ITNs as household rather than health products and increase availability when cash was available. Strong perceptions existed that clinics and salesmen were preferentially selling ITNs to relatives or for profit. Year-round availability of ITNs, clearer promotion and pricing, and improved staff monitoring could reduce complaints [45,46].

Some high-risk households (e.g. widow-led) did not have sufficient funds to purchase ITNs at prevailing prices. While free mass distribution is more difficult to justify in this low-transmission area, a targeted subsidy to make ITNs affordable for those who truly cannot pay, or free distribution to most-at-risk groups is probably necessary to improve coverage rates [34,47,48]. Additional distribution through local shops, where poorer women can get credit, would also increase access [43,48].

Table 1 shows the price of an ITN was only slightly more than the average cost of treating one malaria episode. However, participants noted that emergency funds were easier than prevention funds to mobilise. It is also worth noting that non-owners reported slightly higher average malaria treatment prices (Table 1). This might indicate either the operation of informal credit mechanisms or respondent bias. As reported ITN prices were the same for both groups, more research on informal financing mechanisms may be warranted. However, authors found that money was as sensitive an issue as gender in rural areas. Since this study was conducted, foreign funding for malaria control in Afghanistan has increased dramatically. Mass distribution of LLINs is coordinated by HNI-TPO through a greatly expanded clinic-based system. ITNs continue to be subsidized rather than provided free to users and findings reported here are still applicable.

### ITN coverage and usage

Three key issues raised by ITN owners were seasonality, sleeper prioritization, and poor quality re-treatment. Most participants used ITNs in summer. This corroborates an earlier clinic-based case-control study of ITN effectiveness against malaria in Nangarhar, showing that despite high summer and autumn temperatures this was when people used nets most [18]. Fortunately, this is the peak malaria transmission season [17]. Many who did not use ITNs reported sleeping wrapped in their water-soaked *chadors *to keep cool. In emergencies, *chadors *treated with the repellent insecticide *permethrin *have provided some malaria protection [22]. Soaking in water could limit the effectiveness of insecticide-treated *chadors *unless rendered wash-resistant through newer formulations.

Prioritisation of women and children under ITNs is a key health message emphasized by ITN implementers in Afghanistan [19]. Most families automatically prioritised children, and to a lesser extent women, so messages could focus on youngest children and pregnant women as those at greatest potential risk. With the new donor focus on gender issues and women's capacity-building in Afghanistan, there is more scope for improvement in this area than when this study was conducted.

A widely held perception that the quality of insecticides used to re-treat ITNs had deteriorated was attributed to implementers over-diluting insecticide. In reality, the type of insecticide used had changed. Initially HNI-TPO used *permethrin*, but as this became harder to obtain the agency switched to *lambda-cyhalothrin *and *deltamethrin*. These alphacyanopyrethroids are more toxic but less repellent than *permethrin*, possibly giving the impression implementers had tampered with insecticide [49-51]. If people continue to perceive that the retreatment insecticide does not work, they would be less likely to have ITNs retreated or recommend that others buy ITNs. Fortunately, with the transition to long-lasting technologies this is less of an issue, as only LLINs are now sold in Afghanistan.

During the malaria eradication era of the 1950s and 1960s, IRS was widely and successfully applied in Afghanistan [52,53]. IRS and effective treatment drugs, as parasites were not yet resistant to chloroquine, led to low malaria prevalence in most parts of the country [54]. Effective IRS campaigns require infrastructure and planning that was not feasible during the Soviet and civil wars [25]. Participants indicated they would favour re-introducing an IRS programme, as it protected all household members, reduced overall mosquito numbers, and was free-of-cost to households. However, the additional technical and coordination requirements often make it more costly than ITNs for implementers [55,56]. As sentinel surveillance data indicates malaria in Afghanistan is declining, coinciding with the scale-up of LLINs and wider use of more effective anti-malarial drugs, a return to IRS appears unwarranted.

### Gender issues

Three gender-related issues could be further addressed. First, the authors found it possible to conduct research among Afghan women, even in Taliban-controlled areas. Teaming a foreign female and Afghan male researcher was a relatively novel approach in these isolated areas. Women wanted to meet 'the foreign lady,' and allowing curiosity to overcome suspicion enabled access to women in their home environment that might not otherwise have been allowed. Such direct engagement could increase bias if not handled sensitively, but worked effectively when combined with findings from other data sources. More could be done to adapt social research methods for conservative, conflict-affected, and insecure environments.

Second, while men had the primary role in household decision-making, information access and purchasing, older women particularly mothers-in-law exerted considerable influence. Efforts could be made to mobilize these women more effectively in health promotion campaigns.

Third, male-focused health education did appear to filter through to women, making this a potential approach for socially-segregated environments. Interviews and FGD results with women corresponded well with quantitative survey findings, even though most survey participants were men.

Issues for further investigation include vulnerability in access to ITNs and the status of widow-led families in post-war Afghanistan, women whose husbands are economic migrants, and landless households. Work has been done on this in Africa, but more research is required to identify how best to target and support these high-risk households in Afghanistan [36,42,45,57]. Additional research could explore delivery strategies and cost-effectiveness of ITNs in areas of relatively low malaria endemicity or whether ITNs should form the basis of a malaria elimination strategy - important issues that were not addressed in this study.

Conditions in parts of rural Afghanistan have not changed greatly since this research was conducted. The south and east of the country are, if anything, more insecure under allied occupation and as a result INGOs have less freedom to operate than did the authors in 2000. Health funding and infrastructure have greatly improved over the decade since the Taliban last held power. However, availability of health services in remote communities remains variable. While women have greater freedom of movement and better access to health care and information than under the Taliban, they still face many unchanged financial and social barriers. This paper provides an indication of health-related constraints and potential approaches should support for the Afghan government deteriorate or the Taliban once again gain control in Afghanistan.

## Competing interests

The authors declare that they have no competing interests.

## Authors' contributions

NH designed and conducted interviews and household survey, conducted analysis, and drafted the manuscript. AS moderated interviews and FGDs, transcribed and translated data, assisted in analysis and critically reviewed the manuscript. CJ supervised focus groups, analysed focus group data, and critically reviewed the manuscript. MR conceived of the study, participated in its design and coordination and critically reviewed the manuscript. All authors read and approved the final manuscript.
